# Laparoscopic intragastric resection of gastric synovial sarcoma: report of the first ever case with video demonstration

**DOI:** 10.1186/s12957-021-02172-y

**Published:** 2021-03-01

**Authors:** Matteo Rivelli, Eduardo Fernandes, Cristian Conti, Laura Bernardoni, Sara Pecori, Sara Cingarlini, Corrado Pedrazzani

**Affiliations:** 1 Unit of General and Hepatobiliary Surgery, Department of Surgical Sciences, Dentistry, Gynecology and Pediatrics, Verona University, Verona, Italy; 2 Division of Minimally Invasive, General and Robotic Surgery, University of Illinois at Chicago, Chicago, USA; 3 Digestive Endoscopy Unit, The Pancreas Institute, Verona University and Hospital Trust, Verona, Italy; 4 Pathological Anatomy Section, Department of Diagnostics and Public Health, Verona University and Hospital Trust, Verona, Italy; 5 Oncology Section, Department of Oncology, Verona University and Hospital Trust, Verona, Italy; 6 Unit of General and Hepatobiliary Surgery, University Hospital “G.B. Rossi”, Piazzale “L. Scuro” 10, 37134 Verona, Italy

**Keywords:** Synovial sarcoma, Gastric sarcoma, Laparoscopy, Intragastric resection, Organ-preserving surgery

## Abstract

**Background:**

Synovial sarcoma (SS) is a rare soft tissue tumor. Among different anatomical locations where it can be found, gastric localization is a very uncommon one. Based on soft tissue sarcoma guidelines, complete tumor excision is considered the main treatment approach. Depending on size and localization of the tumor, both wedge and major gastric resections have been performed in the past for the treatment of this condition.

**Case presentation:**

We present the case of a 43-year-old woman who underwent a laparoscopic intragastric excision of a gastric 10-mm SS located nearby the esophagogastric junction. Pathology examination confirmed the presence of a SS. The resected specimen confirmed margin-free excision of a monophasic spindle cell neoplasm invading the submucosa and presenting the rearrangement of SS18 gene at fluorescence in situ hybridization (FISH). No adjuvant treatment was offered, and 18 months after surgery, the patient was alive and disease free.

**Conclusions:**

This represents the first case reported in literature of a laparoscopic intragastric resection for a gastric SS. This approach allowed to obtain a full thickness radical tumor resection with the advantages of minimally invasive and organ preserving surgery.

**Supplementary Information:**

The online version contains supplementary material available at 10.1186/s12957-021-02172-y.

## Background

Synovial sarcoma (SS) is a rare malignant tumor derived from mesenchymal tissue and has an incidence of 8 to 10% among all soft tissue sarcomas [1]. Even though it is called “synovial,” its real origin is still unknown as there are no evidences of direct differentiation from synovial tissue.

It occurs primarily in the extremities of arms and legs, often in proximity to joint capsules and tendon sheaths, but it has been rarely observed in the head and neck region, mediastinum, blood vessels, heart, liver, and in gastrointestinal tract [2–8].

When located in the gastrointestinal tract, it can be easily mistaken for a gastrointestinal stromal tumor (GIST). What sets these two entities apart is the presence of an SSX-SS18 chromosome translocation [9].

Interestingly, only 40 cases of SS have been reported to have a gastric location [10]. As per virtually all soft tissue tumors, surgical resection with negative margins represents the mainstay of therapy [11, 12]. Major gastric resections as well as wedge or tumor resections, mainly by open surgery, have been performed in the past depending on the size and location of the tumor [10, 13].

We present the first case of laparoscopic intragastric surgery for the treatment of a gastric SS.

## Case presentation

**Fig. 1:**
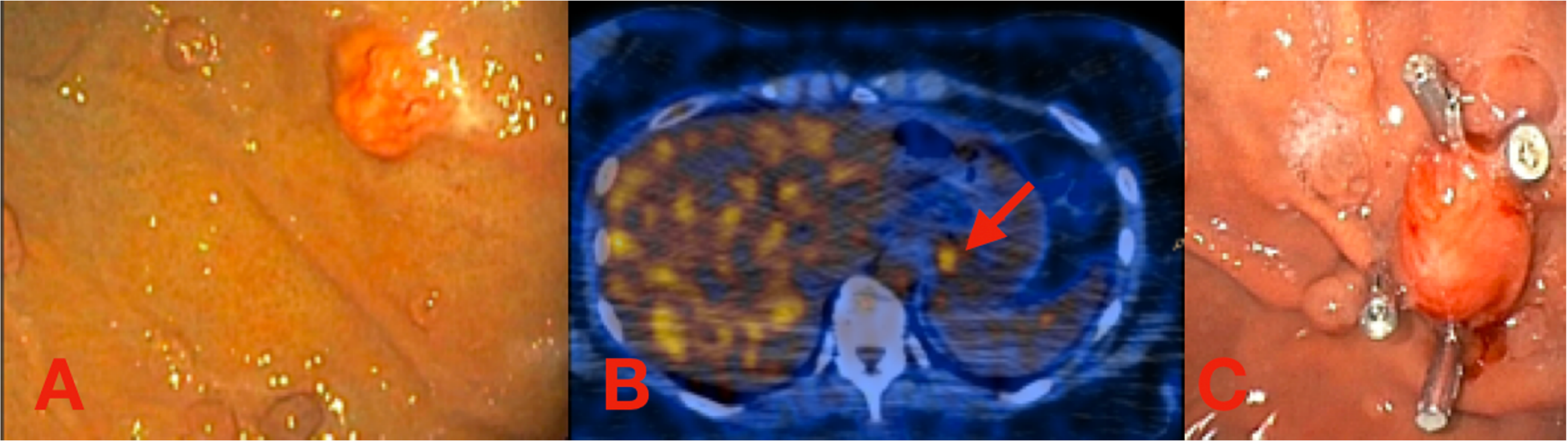
**a** Diagnostic esophagogastroduodenoscopy. **b** PET-CT scan showing a single gastric uptake (red arrow). **c** Preoperative esophagogastroduodenoscopy with clip positioning

A 43-year-old woman was referred to our Tertiary Care Center for a submucosal ulcerated lesion of the upper third of the stomach. Her past medical history was unremarkable, with the exception of a 1-year history of epigastric pain and dyspepsia which failed to improve on standard PPI treatment. Esophagogastroduodenoscopy (EGDS) showed atrophic gastritis with multiple amartomatous polyps and a 10-mm submucosal round-shaped lesion with central ulceration located in the upper third of the stomach in proximity of the esophagogastric junction (Fig. 1a). Multiple biopsies with immunohistochemical analysis and fluorescence in situ hybridization showed a rearrangement of SS18 gene, indicative for synovial sarcoma.

Preoperative staging work-up was completed via chest-abdomen computed tomography (CT) which failed to identify the lesion and a fluorine-18-labeled fluorodeoxyglucose positron emission tomography (18F-FDG PET/TC) which demonstrated the presence of a single metabolically active mass in the upper third of the stomach (Fig. 1b).

Given size and location of the tumor near the cardial region, a laparoscopic intragastric approach was used. This approach was employed also based on the fact that no formal lymphadenectomy was deemed necessary. The day before surgery, a further endoscopic evaluation with clip tumor marking was carried out to facilitate intraoperative tumor identification (Fig. 1c).

**Fig. 2:**
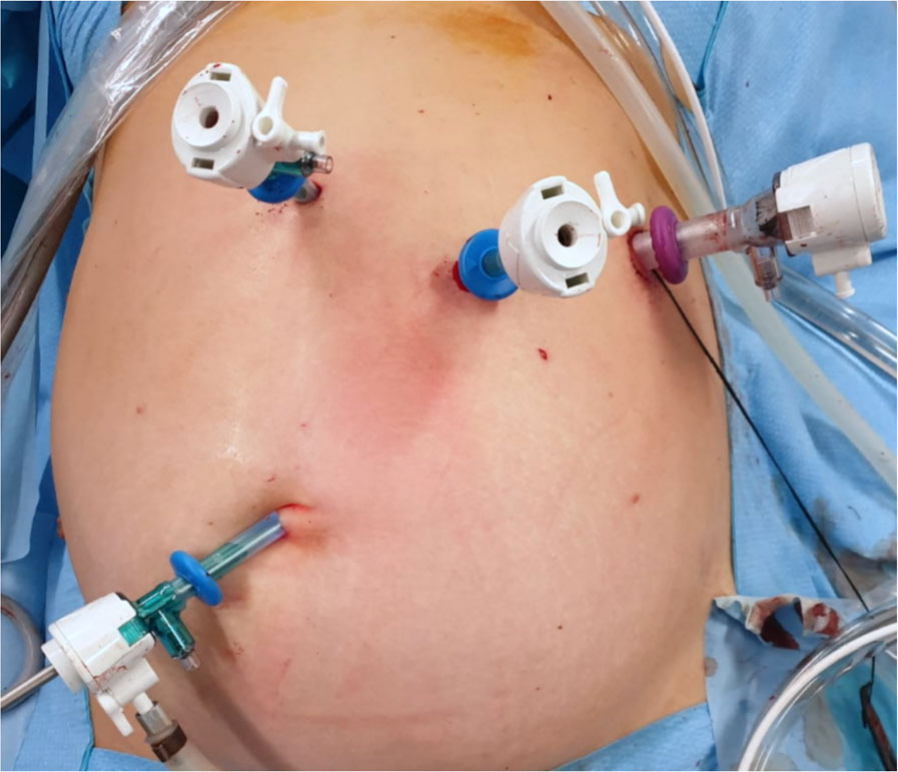
Trocar positioning


The patient was placed on the operating table in the supine position with parted legs, 20° reverse Trendelenburg, and slight left-side tilt. A 5-mm camera was used. A 5-mm trocar was placed at the umbilicus, and further, two 5-mm balloon trocars and one 12 balloon trocar were placed in the mid-left upper quadrants of the abdomen considering the position of gastric body in relation to the abdominal wall (Fig. 2). This multiport approach allowed us to perform a full abdominal cavity exploration, an accurate intragastric port placement, the use of an endoscopic linear stapler for a complete full thickness tumor resection, and an easy and safe closure of gastrotomies (Video).

**Additional file 1.** Supplemental Digital Content 1.

Postoperative course was uneventful. Soft diet was resumed on postoperative day 1, and patient was discharge on postoperative day 3.

**Fig. 3:**
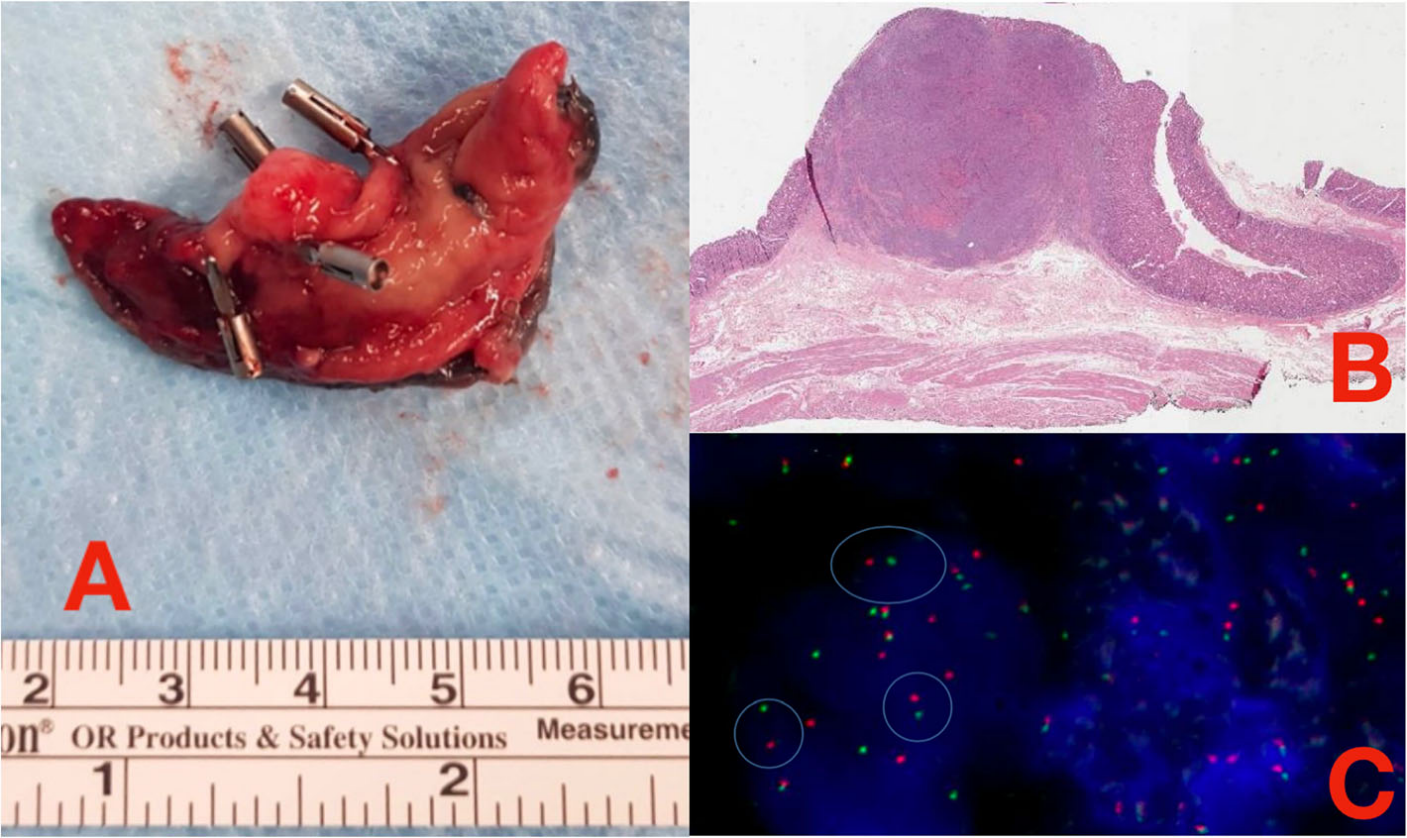
**a** Surgical specimen. **b** Hematoxylin eosin strain showing the whole section of the resected tumor. **c** FISH analysis showing SS18 gene rearrangement

Histopathology examination confirmed the presence of a monophasic spindle cell neoplasm. Four out of ten mitoses on high magnification field was shown, no necrosis was present, and lympho-vascular invasion was seen. The tumor involved the submucosa. Circumferential and deep margins were tumor free. Fluorescence in situ hybridization established the rearrangement of SS18 gene confirming the diagnosis of SS (Fig. 3a, b, c).

No adjuvant treatment was offered, and 18 months after surgery, the patient is alive and disease free.

## Discussion

Primary gastric SS is an exceptionally rare disease. The first case was described in 2000, and 40 cases have been reported since [9, 10].

At endoscopic examination, gastric SS resembles a GIST tumor, though diagnosis should be suspected when a white submucosal plaque-like lesion with central depression is observed [9, 14].

On hematoxylin and eosin staining, histology demonstrates a similar pattern or mono or biphasic spindle cell in both SS and GIST tumors. On immunohistochemistry, however, c-kit positivity is lacking in gastric SS, and the fusion products of the SS18 gene combined to either SSX1, SSX2, or SSX4 gene are found [15]. Three histological variants of SS have been described: (1) monophasic, (2) biphasic, and (3) a “poorly differentiated” subtype. The monophasic and biphasic subtypes represent the 89% and 8% of the cases, respectively. The “poorly differentiated” subtype has been observed only in one case [10, 13, 15, 16].

With regard to treatment, no definite guidelines are available for gastrointestinal SS. Based on NCCN and ESMO guidelines for soft tissue sarcomas, complete tumor resection is considered the main goal of surgery and no lymphadenectomy is indicated [11, 12].

In previous series, different surgical approaches have been described in 29 cases: a major gastric resection was performed in 16 cases, and a wedge or tumor resection was carried out in 13 cases [9, 10, 13–15, 17–27]. A laparoscopic approach was employed in 7 cases [13, 18, 23–27].

In our case, despite the small size of the lesion, a proximal or total gastrectomy would have been required to achieve tumor clearance due to its location nearby the esophagogastric junction. The choice of a laparoscopic intragastric approach allowed us to perform a complete tumor resection within the objective of organ preservation. This approach has been successfully performed in GISTs as well as other tumors located on the posterior wall of the upper third of the stomach when no lymphadenectomy is required [28, 29]. This approach provides minimal patient discomfort in the early postoperative course and avoids post-gastrectomy short- and long-term complications.

## Conclusions

This is the first case reported in literature of laparoscopic intragastric resection for a synovial sarcoma of the stomach. This approach allowed to obtain a full thickness complete resection of the tumor with the preservation of the stomach, hence combining the advantages of minimally invasive and organ-preserving surgery.

## Data Availability

Data sharing not applicable to this article as no datasets were generated or analyzed during the current study.
